# Familial episodic ataxia in lambs is potentially associated with a mutation in the *fibroblast growth factor 14* (*FGF14*) gene

**DOI:** 10.1371/journal.pone.0190030

**Published:** 2017-12-18

**Authors:** K. E. Dittmer, R. D. Jolly, I. G. Mayhew, A. L. Ridler, A. Chernyavtseva, D. J. Garrick, H. T. Blair

**Affiliations:** 1 Institute of Veterinary, Animal and Biomedical Sciences, Massey University, Palmerston North, New Zealand; 2 Department of Animal Science, Iowa State University, Ames, Iowa, United States of America; The University of Melbourne, AUSTRALIA

## Abstract

Familial episodic ataxia of lambs is a congenital transient autosomal dominant disorder of newborn lambs, with varying expressivity. Affected lambs show episodes of an asymmetric ataxic gait, base-wide extensor hypertonia of the thoracic limbs and flexor hypertonia of the pelvic limbs. The aim of the study was to determine the genetic variant causing familial episodic ataxia in lambs. Using whole genome sequencing of two half-sib affected lambs, their sire, and their two normal dams, a heterozygous C>T transition at OAR10:77593415 (Oar_v3.1) in exon 1 of the *fibroblast growth factor 14* (*FGF14)* gene (c.46C>T) was identified. The c.46C>T transition resulted in a premature stop codon at position 16 of the 247 amino acid FGF14 protein (p.Q16*). PCR and Sanger sequencing was used to genotype an additional 20 clinically affected animals, demonstrating all lambs carried the c.46C>T variant but 1 clinically more severely affected inbred lamb was homozygous (TT). A further 11 unrelated normal ewes were positionally sequenced, none of which had the variant, while in 18 lambs of unknown status born over 2 years of breeding trials six lambs were found to have the c.46C>T variant, likely clinically unidentified heterozygotes due to the variable expressivity, while 12 did not. In conclusion, familial episodic ataxia of lambs is potentially associated with a c.46C>T variant in the *FGF14* gene. Further research is required into the mechanism behind the apparent recovery of lambs.

## Introduction

There are many inherited syndromes characterized in part by cerebellar ataxia. In human patients these are classified by: the mutated gene in question; by the gene action being autosomal dominant, autosomal recessive, sex-linked or mitochondrially inherited; and then further into sub-types by reference to certain clinical features [1]. There are over 50 disorders classified as autosomal dominant spinocerebellar ataxias (SCA) and seven as autosomal dominant episodic ataxias (EA). The former mainly present as slowly progressive ataxia in young children, but later onset cases do occur. Cases of EA have an earlier onset and also differ by their episodic nature, a feature not characteristic of the SCAs, although some cases may begin with episodic manifestations [2]. Clinical features of the SCAs and EAs are variable, likely as a result of the difficulty in accurately diagnosing each disease due to difficulties in characterising the phenotype, due to varying gene expressivity, or due to there being different mutations of the same gene.

This paper concerns a familial episodic ataxia in lambs first discovered in New Zealand in 2009, that is inherited as an autosomal dominant trait with varying expressivity [3]. At birth, some affected lambs were noted to have a head and neck extended posture and were slow to get to their feet and suckle, after which they became more or less normal. When forced to move, affected lambs had an asymmetric abnormal gait. This consisted of base-wide extensor hypertonia with hypometria of thoracic limbs, and flexor hypertonia with hypermetria of pelvic limbs. In some lambs there was spontaneous and postural nystagmus. After several metres of asymmetric ataxic gait they would fall to one side while struggling and sometimes would assume a sitting posture. Recovery usually occurred within a few minutes. As lambs aged, it became more difficult to elicit clinical episodes of dysfunction and by 3–6 months they appeared normal. No histological lesions have been detected in affected lambs. To the authors’ knowledge, a similar episodic condition has not been described in sheep.

SCA have been described in dogs but to our knowledge no other domestic species [4–9]. The clinical features and lesions of SCA in dogs differ to those in our study. Canine hereditary ataxia in Old English Sheepdogs and Gordon setters is a slowly progressive cerebellar ataxia associated with autophagosome accumulation in cerebellar purkinje cells due to a variant in the *RAB24* gene [5]. In contrast Finnish hounds with a progressive early onset cerebellar ataxia have shrinkage of the cerebellum and marked loss of Purkinje cells due to a *SEL1L* variant [7]. A *KCNJ10* variant is associated with SCA in Fox and Russell terriers, which present clinically with hypermetria and in some cases neuromyotonia and seizures [6]. While in Coton de Tulear dogs, also with cerebellar ataxia and intention tremors and lacking histologic lesions, a variant in *GRM1* has been found [4]. Hypermetria and cerebellar ataxia ultimately leading to inability to walk by 4 months of age is described in Italian Spinone dogs due to a *ITPR1* variant [9].

The aim of this study was to determine the causative mutation for familial episodic ataxia of lambs by whole genome sequencing and compare the disorder with similar human ataxic disorders.

## Materials and methods

### Animals, DNA and RNA extraction

Two rams (A & B), which had been identified as sires of affected lambs on two different farms, were acquired and bred over 2 successive years to 50 unrelated, mixed age, New Zealand Romney-type ewes as described in Mayhew *et al*. (2013). Ram A was of Romney breed, whereas ram B was an East Friesian-Romney cross, both rams having been bred by the same ram breeder. In addition to the outcross matings to unrelated ewes, ram B was mated to four of his own clinically normal daughters. All breeding and experimental procedures were undertaken with approval of the animal ethics committee of Massey University (New Zealand) and conformed to “The Code of Ethical Conduct for the Use of Animals for Teaching and Research” as approved under the New Zealand Animal Welfare Act 1999. Blood from the sires, dams, and the lambs born over the 2 years of the breeding trial was collected by jugular venipuncture into lithium heparin vacutainer tubes, and stored at -20°C until further processing. Phenotype was determined as described in Mayhew et al., 2013.

DNA to be used for whole genome sequencing was extracted from the stored blood of one sire (ram A), two of his affected progeny, and the two dams of the same affected progeny, using the MagAttract HMW genomic DNA extraction kit (Qiagen GmbH, Germany) as per the manufacturer’s instructions. This was followed by sample clean-up using the Genomic DNA Clean and Concentrate-10 kit (Zymo Research Corp., Irvine, CA, USA) as per the manufacturer’s instructions. The DNA concentration was determined using the Life Technologies Qubit dsDNA BR assay kit with the Qubit 2.0 fluorometer (Life Technologies Corp., Irvine, CA, USA), and the purity by determining the OD260:280 and OD260:230 ratios using the Nanodrop spectrophotometer (Thermo Fisher Scientific Inc. Waltham, MA, USA). Finally DNA integrity was determined by running 5 μL of the DNA sample on a 1.5% agarose gel containing ethidium bromide with visualisation of a single high molecular weight band using an ultraviolet transilluminator.

DNA to be used for genotyping was extracted from stored blood of 20 affected lambs, Ram B, 18 lambs of unknown status born over 2 years of breeding trials for this defect, and 11 unrelated normal ewes (dams in the breeding trials) using the DNeasy Blood and tissue kit (Qiagen GmbH, Germany) as per the manufacturer’s instructions.

Ovine DNA stored at -20°C from 71 control sheep which consisted of 5 Texel, 25 Corriedale, 8 Perendale, 17 Romney, 4 Coopworth, and 12 Wiltshire sheep were used for validation of the mutation.

A known affected animal (Affected 3), a ram sired by ram B and born during the breeding trials, was humanely euthanased and cerebellum for RNA isolation was collected within 30 min of euthanasia, cut into small pieces, snap frozen in liquid nitrogen and stored at -80°C until processing. Total RNA was extracted from the cerebellum using the Omega Bio-tek E.Z.N.A. Total RNA kit (Omega Bio-tek Inc., Norcross, GA, USA) as per the manufacturer’s instructions. RNA and DNA concentrations were measured (to check for absence of DNA) using the Qubit^®^ 2.0 Fluorometer with Qubit^®^ RNA HS, and DNA HS Assays (Life Technologies Corporation, Carlsbad, CA USA). In addition the RNA integrity was assessed by running 10 μL of the RNA sample on a 1.5% agarose gel containing ethidium bromide with visualisation of two sharp 28S and 18S bands using an ultraviolet transilluminator.

### Whole genome sequencing

The high quality, high molecular weight DNA, minimum 5 μg at greater than 20 ng/μL, was sent to the sequencing facility, New Zealand Genomics Ltd, Otago, New Zealand, where a fragment library was prepared using the Illumina TruSeq DNA PCR free library preparation kit (Illumina Inc., San Diego, CA, USA) with a 550 bp insert size. Two lanes of paired-end reads (2 x 100 bp) were then obtained using an Illumina HiSeq 2000 machine (Illumina Inc., San Diego, CA, USA). The raw reads were filtered and trimmed to remove adaptor sequences and low quality reads using FastQC (http://www.bioinformatics.bbsrc.ac.uk/projects/fastqc/) and SolexaQA dynamic trim (P = 0.05) [10].

### Alignment, variant detection and filtering

The fastq files of the paired end reads, designated R1 and R2, were concatenated, followed by alignment to the reference sheep genome (Oar_v3.1) using BWA-MEM 0.7.12 [11] with–M (marks shorter split hits as secondary) and–R (read group ID attached to every read in output). The resulting SAM file was sorted, converted to BAM format, and duplicates marked in the BAM files using Picard tools 1.136 (https://broadinstitute.github.io/picard). The sequences have been uploaded to the NCBI sequence read archive (SRA) with the project number PRJNA323663, experiments SRX1086093, SRX1806233, SRX1806234, SRX1806235 and SRX1806236.

Variants were called using GATK 3.4–46 HaplotypeCaller in DISCOVERY genotyping mode, using emitRefConfidence GVCF, variant_index_type LINEAR, and variant_index_parameter 12800 [12]. Otherwise, default parameters were used. Joint genotyping was performed by merging the individual g.vcf files for each animal using GATK GenotypeGVCFs mode with the default parameters, and a minimum confidence threshold for calling and emitting variants of Phred quality score of 30 (https://www.broadinstitute.org/gatk/guide/tooldocs). Read coverage was determined using GATK DepthOfCoverage mode.

Genetic variant annotation and effect was predicted using SnpEff 4.1 and reference genome Oar_v3.1.79 [13]. The results were then filtered using SnpSift in order to list the variants that resulted in a stop gain, stop lost, frame shift, or splice site variant, considered to be high impact variants, that were heterozygous in ram A and his 2 affected offspring, but homozygous wild type in the 2 unrelated ewes that were the dams of the 2 affected offspring [14].

Each variant was searched for in the *Ovis aries* 3.1 genome at either the Ensembl database or the European Variant archive (www.ensembl.org or http://www.ebi.ac.uk/eva/). In addition, the function of each gene, or its likely involvement in human disease was determined by searching in Online Mendelian Inheritance of Man (http://www.omim.org/) and National Center for Biotechnology Information, U.S. National Library of Medicine (https://www.ncbi.nlm.nih.gov/gene).

### PCR genotyping and Sanger sequencing of affected animals

The putative mutant variant was genotyped by positional sequencing in 20 affected animals, 18 clinically non-affected animals, 11 unrelated dams of the clinically affected animals, and Ram B using PCR followed by Sanger sequencing. Primers were designed using Geneious 8.1 (Biomatters Ltd., Auckland, NZ) and the Primer 3 algorithm to span the variant, with a forward primer sequence of 5’ TCAGCATCTGAG 3’ and a reverse primer sequence of 5’ TCCAGGTGGTCCAAGGATCCAGGCTTGAA 3’, resulting in a 460 bp fragment. The PCR mix contained 1 X PCR buffer, 1.5 mM MgCl_2_, 0.2 mM each dNTP (Fisher BioTec Ltd. Wembley, WA, Australia), 0.2 mM each primer, 1 unit Taq-Ti DNA polymerase (Fisher BioTec Ltd., Wembley, WA, Australia) and 2 μL DNA, made up to 25 μL with H_2_O. In each PCR run a negative control containing 2 μL H_2_O was included to check for the presence of contaminants. The PCR conditions were as follows: 95°C for 10 minutes followed by 35 cycles of 95°C for 30 s, 63°C for 30 s, 72°C for 1 minute, with a final extension of 72°C for 5 minutes and then chilled at 4°C using an Applied Biosystems Veriti Thermal Cycler (Applied Biosystems Inc., CA, USA). The PCR products were analysed on a 1.5% agarose gel containing ethidium bromide and visualised using an ultraviolet transilluminator to check for successful amplification. This was followed by purification of the remaining PCR product using the QIAquick PCR purification kit (Qiagen GmbH, Germany). The amplicon with the PCR primers was sent to the Massey University Genome Service (Massey University, Palmerston North, New Zealand) and subjected to automatic dye-terminator cycle sequencing with BigDye Terminator version 3.1 Ready reaction cycle sequencing kit and the ABI3730 Genetic Analyzer (Applied Biosystems Inc., CA, USA). The sequence data was analysed using Geneious 8.1.

The Transcriptor first strand cDNA synthesis kit (Roche Molecular Systems Inc., Penzberg, Germany) was used to synthesise cDNA from RNA extracted from the cerebellum of an affected ram. Each reaction tube contained 600 ng RNA, 2.5 μM oligo(dT), 8 mM RT reaction buffer, 1 mM dNTP, 10 U reverse transcriptase, 20 U RNase inhibitor, made up to 20 μL with RNase-DNase free water. The reaction was performed at 55°C for 30 min, 85°C for 5 min, and then chilled at 4°C using an Applied Biosystems Veriti Thermal Cycler. Primers were designed using Geneious 8.1 and the Primer 3 algorithm to span the variant in the coding sequence, with a forward primer sequence of 5’ CCGTCGAGAGCTCCATCG 3’ and a reverse primer sequence of 5’ CCTGGTGGACATCTTCTCCA 3’, resulting in a 150 bp fragment. The PCR mix, conditions, visualisation and sequencing were as described above.

## Results

Whole genome sequencing of five sheep, namely one sire (ram A), two affected lamb offspring and their two unaffected dams resulted in 192–284 Mbp of sequence data, as summarised in Table 1. Filtering the variants that were heterozygous in ram A and two affected lambs but homozygous wildtype in the two dams, resulted in 336,066 variants, of which 186 were unique non-synonymous (missense) variants (S2 Table), 30 were unique frame shift variants (insertions or deletions), 15 were unique stop gained variants, 5 were unique splice site variants, and 1 was a unique stop lost variant (S1 Table). The frame shift, stop gained/lost and splice site variants were considered to be high impact variants, of which there was a total of 52 variants affecting 29 different genes.

**Table 1:** Summary data of whole genome sequencing using Illumina HiSeq 2000 machine and aligned to the reference sheep genome (Oar3.1) using BWA-MEM.

	Ram A	Affected 1	Affected 2	Dam of affected 1	Dam of affected 2
**Total Reads Mbp**	284	192	199	232	231
**Mean quality score (PF)**	37.4	37.1	36.8	36.9	36.7
**% ≥ Q30**	93.4	92.4	91.5	91.8	90.9
**Reads mapped %**	84	84	83	84	81
**Mean genome coverage**	12.7	8.6	8.9	10.4	10.1

The data collected on each non-synonymous missense and high impact variant is summarised in S1 and S2 Tables. As illustrated in the S1 and S2 Tables, only 1 variant was in a gene known to cause a similar disease in humans, fibroblast growth factor 14 (*FGF14*). Of the listed variants, with the exception of the variant in *FGF14*, no others were associated with a known human disease, mouse model, gene pathway or function related to the ataxia and neurological signs seen in affected sheep. Twenty-six variants were in uncharacterised proteins, and two of these had known stop or frame shift variants that appeared unrelated to any clinical disease, but due to the uncharacterised nature of the proteins their function was unable to be investigated further.

**Fig 1:**
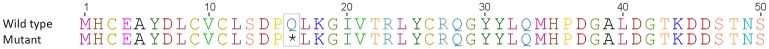
Protein sequence alignment of fibroblast growth factor 14. Protein sequence alignment of wild type and mutant ovine fibroblast growth factor 14, highlighting the Q16X mutation which induces a stop codon. The Q16X mutation was determined after whole genome sequencing using Illumina HiSeq 2000 machine, aligning to the reference sheep genome (Oar3.1) using BWA-MEM, calling variants with GATK 3.4–46, and predicting variant effects with SnpEff 4.1.

Thus, a heterozygous C>T transition at OAR10:77593415 (Oar_v3.1) in exon 1 of the *FGF14* gene (c.46C>T) (Gene ENSOARGG00000004918, transcript ENSOART00000005357, www.ensembl.org) was identified in ram A and the two affected progeny, and considered most likely to be the causative mutation of the clinical syndrome. The c.46C>T transition resulted in a premature stop codon at position 16 of the 247 amino acid FGF14 protein (p.Q16*) (Fig 1). This nonsense mutation was not a known variant in the Ensembl *Ovis aries* database (www.ensembl.org/Ovis_aries) or the European Variant Archive (http://www.ebi.ac.uk/eva/).

**Fig 2:**
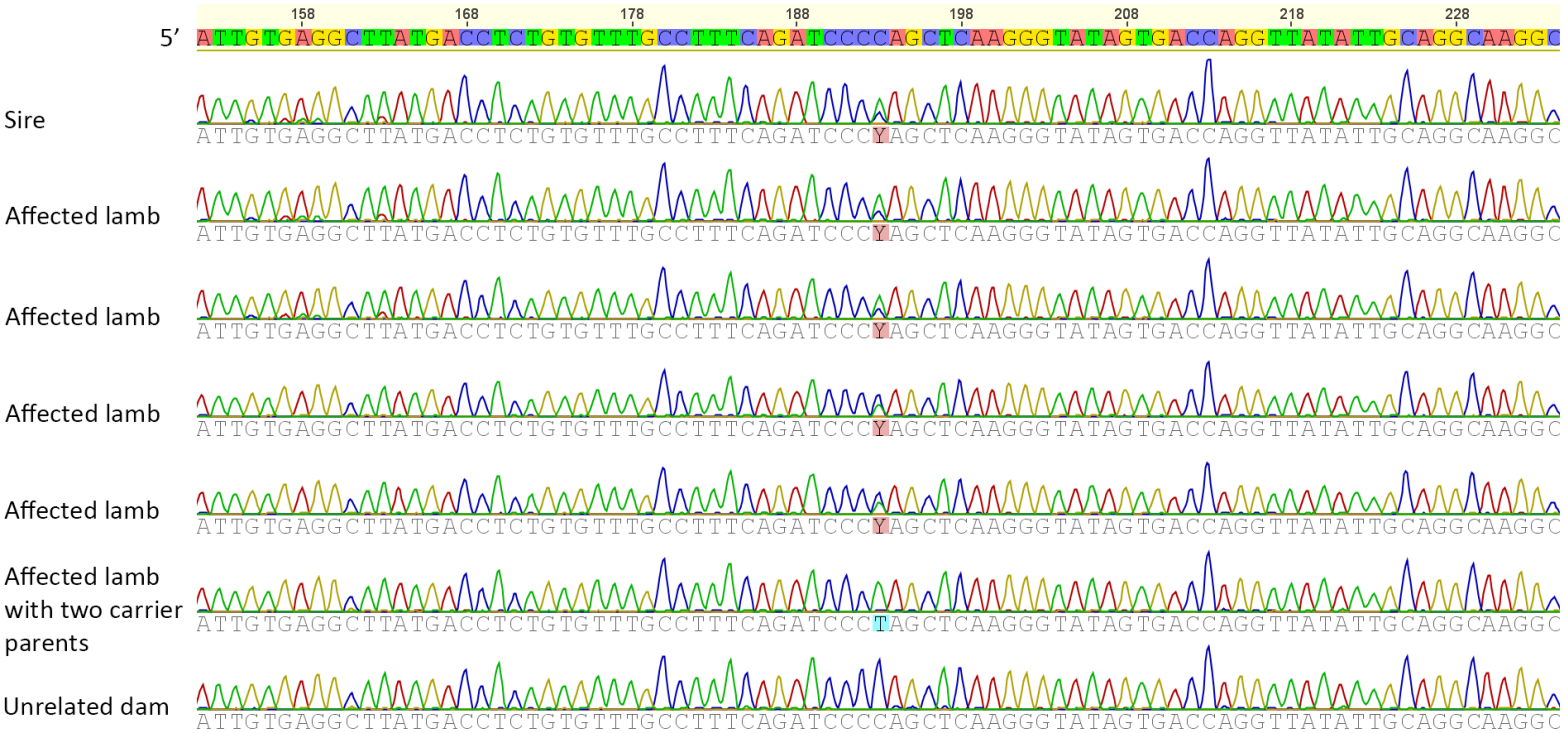
Chromatogram showing the location of the mutation Y: C/T in heterozygous, affected, and unrelated normal sheep. The c.46C>T variant was determined after whole genome sequencing using Illumina HiSeq 2000 machine, aligning to the reference sheep genome (Oar3.1) using BWA-MEM, calling variants with GATK 3.4–46, and predicting variant effects with SnpEff 4.1.

The variant was validated using PCR and Sanger sequencing of DNA from affected lambs, clinically normal lambs born over the 2 years of the breeding trial sired by two rams (A & B), unrelated ewes used as dams in the trial, Ram B, and four older daughters of ram B. The PCR resulted in a single band of the expected size of 460 bp and subsequent sequencing of Ram B and 20 clinically affected lambs resulted in 100% concordance between the presence of this mutation and the familial episodic ataxia of lambs phenotype (Fig 2, Table 2). One of the affected lambs sired by ram B was homozygous for the mutation, this inbred lamb being born to one of the older daughters of ram B. Of the lambs born in the breeding trial that appeared clinically normal, six lambs had the mutation, while 12 were wild type. Of the four older daughters, two were heterozygous for the *FGF14* mutation, including the dam of the homozygous mutant lamb, and two were homozygous for the wild type genotype. None of the unrelated ewes sequenced had the mutation. In addition, none of the 71 control sheep had the mutation.

**Table 2:** Results of Sanger sequencing for the fibroblast growth factor 14 c.46C>T variant in clinically affected, clinically normal and unrelated normal sheep.

Phenotype	Genotype	Number of sheep
**Clinically affected**^a^	C/T	19
	T/T	1
**Clinically normal**^b^	C/T	6
	C/C	12
**Ram B**	C/T	1
**Unrelated normal dams**	C/C	11
**Control sheep**	C/C	71

^a^ T/T genotype inbred lamb sired by ram B mated to one of his older daughters

^b^ includes 4 older daughters of ram B;

The mutation was further validated using RNA extracted from the cerebellum of a clinically affected animal (Affected 3) followed by PCR and Sanger sequencing. The PCR resulted in a single band of the expected size of 150 bp and subsequent sequencing confirmed the heterozygous c.46C>T mutation in the *FGF14* transcript.

## Discussion

Using whole genome sequencing we have discovered a novel nonsense mutation (c.46C>T) in *FGF14* leading to a premature stop codon (p.Q16*) in lambs affected with familial episodic ataxia of lambs. Data from Sanger sequencing showed that all known affected animals carried this mutation. This is further supported by the fact that one more seriously affected inbred lamb, sired by ram B and born to a dam that was also the daughter of ram B, was homozygous for the variant. RNA extracted from the cerebellum of an affected ram showed the presence of the same mutation. In addition, the c.46C>T mutation was absent in 71 sheep of six different breeds. The collective data therefore are consistent with the hypothesis that this c.46C>T variant in *FGF14* is strongly associated with the disease syndrome. However, it should be noted that 6/18 new born lambs carried this variant, but were not observed to be affected at birth. This was considered likely due to the variable expressivity of the disease, the transient nature of the clinical signs, and the field conditions under which the breeding trial was undertaken.

FGF14, also known as FGF homologous factor 4 (FHF4), belongs to a superfamily of conserved FGF proteins (FGF1-23). However, in contrast to other members of the FGF family, FGF11-14 (FHF1-4) are intracellular and do not activate the classical cell surface tyrosine kinase FGF receptors [15–18]. Instead, FGF14 is known to interact with the mitogen activated protein kinase scaffolding protein islet brain 2 (IB2 or MAPK8IP2) [19] and voltage-gated sodium channels [20]. In mice embryos, *FGF14* is widely expressed in the brain and spinal cord in addition to the post-migratory granule cells of the internal granular layer of the cerebellum of mature animals [21]. Homozygous *Fgf14* deficient mice are viable and anatomically normal but smaller in size, and develop ataxia and paroxysmal dyskinesia by one month of age [22].

In humans, mutations in *FGF14* are mostly associated with spinocerebellar ataxia type 27 (SCA27), part of a diverse group of autosomal dominant hereditary ataxias whereby affected individuals develop progressive incoordination [1]. The clinical features of humans with hereditary ataxias and *FGF14* mutations are variable, most cases have been diagnosed in young children ranging in age from 8 months to 5 years [23–26]. However in one Dutch family, where 14 of 21 members of the family were affected, ataxia did not develop until 15–20 years of age, although trembling of the hands developed in childhood [27]. In comparison, lambs with autosomal dominant familial episodic ataxia often were abnormal with an extended head and neck, and extended thoracic limbs in the immediate neonatal period; clinical signs that were possibly triggered by parturition [3]. Episodic ataxia and gait asymmetry could be induced by forced movement but became less severe with age, so that by 3–6 months of age affected lambs were essentially normal [3]. In humans with *FGF14* mutations, the ataxia, dyskinesia and tremors may also be episodic and can be triggered by crying, emotional stress and physical exercise [24, 27].

The phenotype associated with *FGF14* mutations in humans appears to be variable with some individuals clinically diagnosed with either autosomal dominant episodic ataxia [2] or autosomal dominant paroxysmal non-kinesigenic dyskinesia [24] prior to determination of a causative mutation. In at least one family, the disease is thought to have incomplete penetrance [24]. Similar variation in phenotype also appears to occur in lambs with autosomal dominant familial episodic ataxia. Some animals were observed to be affected around the time of birth, and some at the time of tail docking (around 2–3 weeks of age), while others appeared clinically normal, showing the variable expressivity of the disease. In the initial report of the disease, the authors suggested familial episodic ataxia of lambs had autosomal dominant inheritance with incomplete penetrance [3], however we believe this was an incorrect usage of the term incomplete penetrance and suggest variable expressivity would be a more appropriate description. In the experimental trial reported by Mayhew *et al*. 2013 the lambing paddocks were checked only twice daily, therefore it is likely that lambs with transient clinical signs could have been missed and classified as clinically normal, as the transient nature of the familial episodic ataxia in affected lambs made correct classification challenging. The recording of 6/18 new born lambs that were not observed as affected at birth under the field conditions of the breeding trial, but carried this variant, is the expected proportion of “clinically normal” but affected lambs suggested by Mayhew *et al*. 2013. Conversely, the lamb homozygous for the c.46C>T mutation was clinically more severely affected than other affected lambs, with head and neck hyperextension (stargazing posture), tetraplegia, and no eyeball control; that lamb died 24 hours after birth.

Alternatively it should be considered that one of the other high impact or non-synonymous missense variants could instead be associated with familial episodic ataxia of lambs. However, only 1 of the variants was in a gene known to cause similar disease in humans (*FGF14*), while the other variants were either in genes not associated with gene pathways or diseases related to hereditary ataxias (S1 and S2 Tables) or in uncharacterised proteins whose function could not be identified further. Therefore, none of the other variants were considered likely candidates for familial episodic ataxia of lambs.

Three of the reported mutations in *FGF14* in humans with SCA27 involve heterozygous single-base mutations, two single-base pair deletions (c.487delA and c.10delC) [28, 29] and a missense mutation (T145C) [27]. Other reported mutations in humans involve larger deletions, translocations and breakpoint events involving the *FGF14* gene [23–26]. However, the scale of the mutation does not appear to affect the clinical presentation of the disease. The mutation (Q16X) associated with autosomal dominant familial episodic ataxia results in substantial truncation of the 247 amino acid wild type protein, which likely has a significant effect on protein function.

The spectrum of neurological expressions exhibited in diseases with *FGF14* mutations suggests that FGF14 is important for normal functioning of multiple areas of the brain and not just the cerebellum [30]. FGF14 interacts with the pore-forming α-subunit of voltage gated sodium channels (Na_v_), where it controls resurgent current and repetitive firing in Purkinje neurons by facilitating the reopening of the Na_v_ channels [20, 30–32]. Mutant FGF14 does not interact directly with the Na_v_ α subunits, but instead disrupts wild-type FGF14 by preventing it from interacting with the Na_v_ α subunits thus altering neuronal excitability [30, 32, 33]. This disruption of wild-type FGF14 function could perhaps explain the variable expressivity, if one normal gene was able to produce enough wild-type protein to function normally. In addition, *Fgf*^*-/-*^ mice show impaired hippocampal synaptic transmission, particularly presynaptic abnormalities, perhaps explaining in part the learning deficits seen in affected mice and some human cases of SCA27 [16].

Many RNAs containing premature stop codons undergo nonsense mediated decay to protect the cell from the effects of abnormal RNAs [34], however there are some genetic diseases, such as Duchenne muscular dystrophy, where the truncated protein retains some function [35]. A similar effect may occur in Familial episodic ataxia of lambs where the heterozygous c.46C>T variant was detected in RNA extracted from the cerebellum of an affected animal. This would also fit with the proposed pathogenesis for the disease in *Fgf*^*-/-*^ mice where the mutant FGF14 protein disrupts the activity of the wild-type FGF14 protein [32].

It is unclear as to how widespread this ovine mutation is within the national flock, but it may be perpetuated in the ram breeding flock via affected females who survive the neonatal period, or those that do not show clinical signs. More important though, are rams such as Ram A and Ram B that were carrying the mutation and which survived by being or becoming clinically normal. In an extensive farming situation where lambs are not closely observed pre-weaning, it is likely that many lambs could transiently express the phenotype and not be detected as being affected with the disease. In the two proband flocks initially investigated, and sired by such clinically normal rams, there were significant neonatal losses associated with the disorder [3]. Under normal breeding conditions, rams may sire 200 or more lambs a year so the financial loss could be considerable. Therefore, genetic testing of animals in the ram breeding flock may be warranted.

A major point of difference between this ovine disorder and human cases of *FGF14* mutations is that clinical signs of the latter remain for the lifetime of the individual and in some may became progressively more severe with time. In contrast, in lambs, manifestation is in the new born, which then rapidly grow out of the episodes (Mayhew *et al*. 2013). As such, and despite being the only naturally occurring animal model of SCA 27, it is unsuitable for pharmaceutical trials, but could be suitable to investigate variations in transcription (splicing) and expression associated with transitions from embryonic to neonatal to juvenile life stages.

## Conclusions

Familial episodic ataxia of lambs is strongly associated with a c.46C>T variant in the *FGF14* gene. This disease of sheep may be analogous to diseases in humans associated with mutations in *FGF14*.

## Supporting information

S1 Table Stop gained and high impact mutations. Stop gained and high impact variants obtained after whole genome sequencing using Illumina HiSeq 2000 machine, aligning to the reference sheep genome (Oar3.1) using BWA-MEM, calling variants with GATK 3.4–46, and predicting variant effects with SnpEff 4.1. The function of each gene, or its likely involvement in human disease was determined by searching in Online Mendelian Inheritance of Man (http://www.omim.org/) and National Center for Biotechnology Information, U.S. National Library of Medicine (https://www.ncbi.nlm.nih.gov/gene).
(XLSX)

S2 Table Non-synomous missense variants. Non-synomous missense variants obtained after whole genome sequencing using Illumina HiSeq 2000 machine, aligning to the reference sheep genome (Oar3.1) using BWA-MEM, calling variants with GATK 3.4–46, and predicting variant effects with SnpEff 4.1. The function of each gene, or its likely involvement in human disease was determined by searching in Online Mendelian Inheritance of Man (http://www.omim.org/) and National Center for Biotechnology Information, U.S. National Library of Medicine (https://www.ncbi.nlm.nih.gov/gene).
(XLSX)
